# The effect of BIS-guided anaesthesia on the incidence of postoperative nausea and vomiting in children: a prospective randomized double-blind study

**DOI:** 10.1186/s12871-024-02610-w

**Published:** 2024-07-09

**Authors:** Michal Frelich, Peter Sklienka, Tereza Romanová, Simona Němcová, Markéta Bílená, Hana Straková, Karolína Lečbychová, Ondřej Jor, Martin Formánek, Filip Burša

**Affiliations:** 1 Department of Anesthesiology and Intensive Care Medicine, University Hospital of Ostrava, 17.listopadu 1790, Ostrava, 708 52 Czech Republic; 2 Department of Intensive Medicine, Emergency Medicine and Forensic Studies, University of Ostrava, Ostrava, Czech Republic; 3 Department of Otorhinolaryngology and Head and Neck Surgery, University Hospital of Ostrava, 17.listopadu 1790, Ostrava, Czech Republic

**Keywords:** BIS monitoring, Children, Postoperative nausea and vomiting, Postoperative pain

## Abstract

**Background:**

Postoperative nausea and vomiting (PONV) is a significant problem following paediatric surgery, and volatile anaesthetics are an important cause of this phenomenon. BIS-guided anaesthesia, by reducing the consumption of anaesthetics, leads to a decrease in PONV in adult patients.

**Study objective:**

Evaluate the role of BIS-guided anaesthesia in reducing the incidence of paediatric PONV.

**Design:**

Prospective, randomized, double-blind study.

**Setting:**

A single center study in university hospital in Czech republic, from June 2021 to November 2022.

**Patients:**

A total of 163 children, aged 3–8 years with ASA I-II who underwent endoscopic adenoidectomy under general anaesthesia were included.

**Interventions:**

In the intervention group, the depth of anaesthesia was maintained to values between 40 and 60 of BIS.

**Main outcome measure:**

The primary outcome was the incidence of postoperative nausea and vomiting during 24 h after surgery.

**Results:**

The use of BIS-guided anaesthesia led to a significant decrease in the incidence of nausea and vomiting compared to the control group [17% vs. 53%; RR (95%CI) 0.48 (0.27–0.86); *p* < 0.001and 16% vs. 34%; RR (95%CI) 0.33 (0.20–0.54); *p* = 0.01, respectively].

**Conclusions:**

BIS-guided anaesthesia decreases the incidence of postoperative nausea and vomiting in children undergoing adenoidectomy.

**Trial registration:**

Clinicaltrials.gov identifier: NCT04466579.

## Introduction

Postoperative nausea and vomiting (PONV) remains one of the most common adverse events after pediatric surgery, with an overall incidence of approximately 20–30% [1, 2]. However, in the case of high-risk procedures (ENT surgery, strabismus repair), the incidence of PONV can reach up to 80% [2, 3]. PONV is distressing for children and their parents and can be the cause of a number of postoperative complications, including dehydration, electrolyte imbalance, surgical wound dehiscence and bleeding [2]. The etiology of PONV is multifactorial, but one of the main causes, especially in the early postoperative period, is exposure to volatile anaesthetics with a dose-response relationship [4, 5]. The BIS (Bispectral Index) monitor, by measuring the depth of general anesthesia, enables a more accurate titration of anesthetics [6]. The BIS monitor operates on the principle of EEG signal processing using a special algorithm that works with bispectral, power spectral and time domain analysis. The result is a unitless number in the range 0-100, with values of 40–60 being recommended for maintenance of general anaesthesia [7]. A previous studies showed that BIS-guided anaesthesia (BIGA) reduces the incidence of PONV in adult patients [6, 8]. The incidence, risk stratification and pathophysiology of PONV are different in children compared to the adult population [2]. Therefore, we designed a prospective, randomized, and double-blind study to investigate whether BIGA reduces the incidence of PONV within 24 h after endoscopic adenoidectomy under sevoflurane anaesthesia.

## Materials and methods

### Study design

**Fig. 1:**
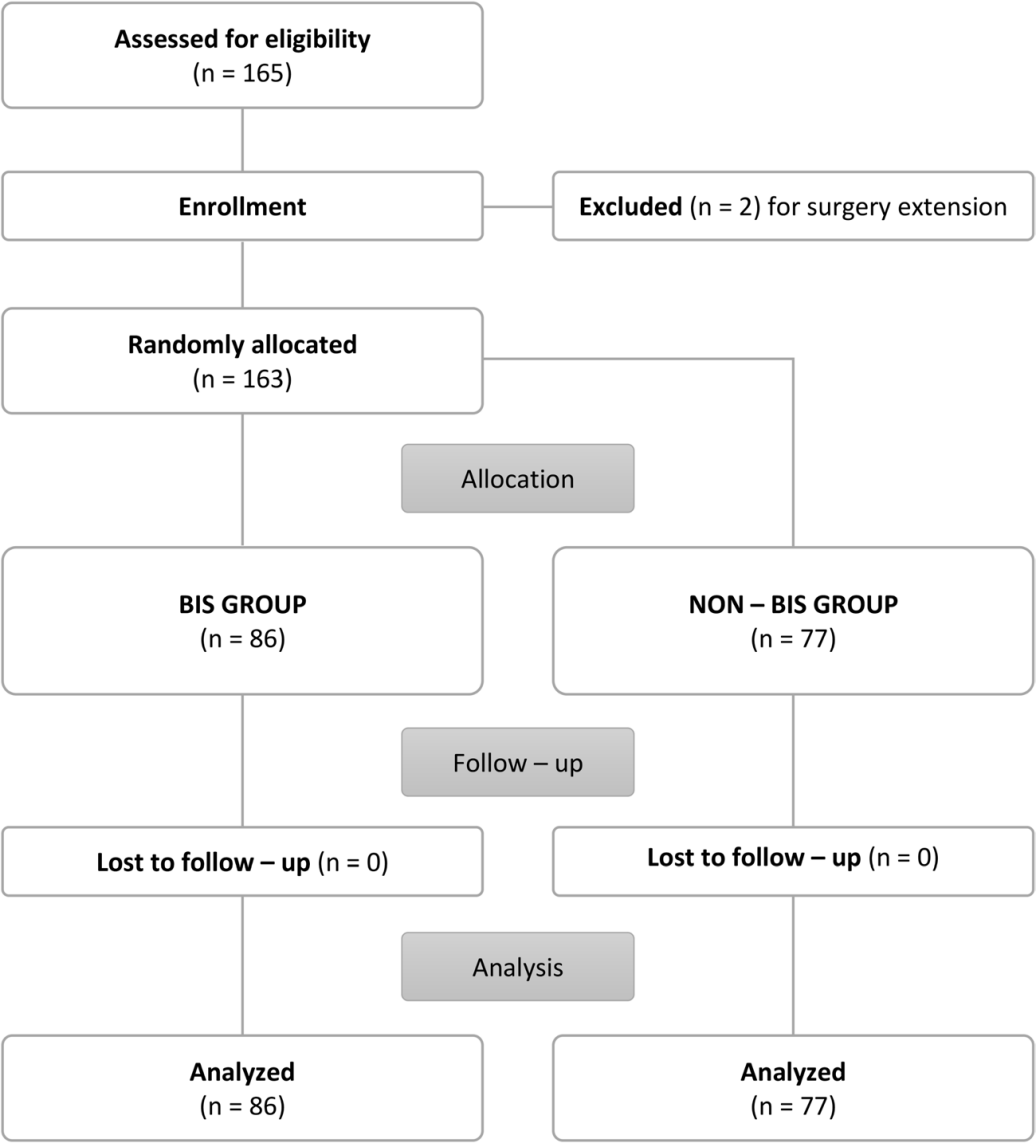
CONSORT flowchart of the study

This prospective, single-center, randomized and double-blind study was approved by the Institutional Review Board (IRB) of the University Hospital Ostrava (18/06/2020, protocol number: 566/2020) and registered on 10/07/2020 (NCT 04466579). Each patient was enrolled in the study after the parents or legal guardians of the participants provided written informed consent. From June 7, 2021, to November 17, 2022, a total of 165 children of either sex who were aged 3–8 years were enrolled in the study. A flowchart of the study is shown in Fig. 1. The study group consisted of children with indications for endoscopic adenoidectomy under sevoflurane anesthesia, and an ASA I-II. The exclusion criteria were an ASA III and higher, previous history of PONV, the presence of study confounders: neurological and gastrointestinal diseases and chronic use of corticosteroids. This study closely followed Good Clinical Practice guidelines and the principles of the Declaration of Helsinki.

### Randomization, intervention, and anesthetic management

All patients received anxiolytic premedication (midazolam 0,5 mg/kg per os) 60 min before the procedure. Upon arrival to the operating theatre, each patient was randomized to either the control (non-BIGA) or the intervention (BIGA) group based on a computer-generated binary code sequence. Anaesthesia was induced by inhalation of sevoflurane, setting the 8% value at the vaporizer in an oxygen and air mixture (FiO_2_ = 0.5). During the induction of anaesthesia, end-tidal sevoflurane concentration (Et _sev_) was recorded every 20s. After a sufficient depth of inhalation anesthesia was achieved (defined by loss of eyelash and central eye position), an intravenous cannula was inserted and sufentanil was administered at a dose of 0.2 µg/kg. All patients received 15 mg/kg i.v. acetaminophen for early postoperative pain management, followed by securing the airway with insertion of laryngeal mask. Mechanical ventilation (MV) was started as synchronized intermittent mandatory ventilation (SIMV) or pressure support ventilation (PSV) according to the patient´s spontaneous breathing activity with the aim of maintaining the end-expiratory carbon dioxide at 35–45 mm Hg. Non-invasive blood pressure, electrocardiogram, heart rate, temperature and pulse oxygen saturation were monitored throughout the surgery and recorded every 5 min. Any adverse events (desaturation, hypotension and bradycardia- defined as a fall below the normal value for a given age group) were recorded. All patients received isotonic crystalloid solution at a rate of 10 ml/kg/hour. Anaesthesia was maintained with sevoflurane. In the intervention group, BIS monitoring (Bispectral Index Monitoring System, MEDTRONIC) was initiated immediately after securing the airway and starting MV, and the depth of general anaesthesia was controlled by titrating sevoflurane with a target BIS value between 40 and 60 throughout the surgery. In the control group, the depth of general anaesthesia was controlled by the anaesthesiologist with the aim of achieving minimum alveolar concentration (MAC) of sevoflurane between 1 and 1.2. In case of patient movement during surgery, sevoflurane concentration on the vaporizer was increased in increments titrated to clinical effect in non-BIGA group. In the BIGA group, sevoflurane was increased to deepen the anaesthesia to a minimum BIS value of 40, and the same recorded in the study proforma. During the procedure, end-tidal sevoflurane concentrations were recorded at 5-minute intervals with the last value at the end of surgery. Total inhalational anaesthetic consumption (measured from the start of anaesthesia to the time the sevoflurane supply is switched off) was obtained directly from anaesthesia delivery system (GE Avance CS2 Pro). Due to the objective of the clinical trial and low-risk study population according to Eberhart´s POVOC score (with wait and see strategy), patients were not administered any prophylactic antiemetic agents [9]. To ensure that the investigators were blinded to patient study allocation, the children’s foreheads were cleaned of electrode marks and the cream was massaged in immediately after BIS monitoring. This procedure was also performed on the children in the control group to ensure that the odour of the cream and redness of the forehead skin did not lead to detection of study allocation. After each patient recovered from general anesthesia (defined by return of the eyelash reflex) and resumed spontaneous breathing, the supraglottic airway device was removed and children were transported to the PACU.

### Postoperative care and data collection in the PACU

After surgery, the patients were continuously monitored for vital signs in the PACU for 60 min. Two well-trained PACU nurses blinded to the group assignment were responsible for evaluating the presence of nausea in all patients with BARF (Baxter Animated Retching Faces) score. The BARF is a validated pictorial scale that uses a series of pictures of faces with expressions of increasing nausea intensity and can therefore be used to assess nausea in young children. The BARF score ranges from 0 (no nausea) to 10 (the most severe level of nausea) and the child, after instruction, simply selects a face with an expression that corresponds to their level of nausea [10]. BARF score was assessed at three timepoints (10 min, 30 min and 60 min during the PACU stay) and value > 4 indicated nausea. Retching (defined as making sound and movement of vomiting without gastric contents) was considered as nausea for the purposes of this study. Each episode of vomiting (defined as the expulsion of gastric contents) was recorded to the study documentation. According to the study protocol, every child with postoperative vomiting (except for patients with retching or one episode of vomiting that lasted for a short period) was administered ondansetron at a dose of 0.15 mg/kg, with a maximum dose of 4 mg i.v. as a rescue antiemetic treatment. Postoperative pain was evaluated according to the FLACC (Face, Legs, Activity, Cry, Consolability) scale at the same timepoints as BARF score. The FLACC is a pain assessment tool that has been validated for use in children aged 2 months to 7 years. This scale contains five categories, each of which is scored from 0 to 2 to provide a total score ranging from 0 to 10 [11]. With a FLACC score > 4, rescue analgesic therapy with metamizole 15 mg/kg i.v. was applied. The need for rescue analgesics or antiemetic agents was recorded in the study documentation.

### Postoperative care and data collection in the standard ward

After 60 min of observation in the PACU, patients were transferred to the ENT department for further postoperative care. A group consisting of two PACU and two ENT nurses assessed the presence of nausea with BARF scale at 2, 4, 6 and 8 h after surgery and recorded every episode of vomiting during 24 h period. The criteria for nausea and vomiting were set in the same way as in the PACU. The research team, as well as the patients and their parents, were unaware of the children’s study allocation.

### Statistical analysis

The power analysis of the study was based on reducing the incidence of PONV in the intervention group. The estimated incidence of PONV in the control group was determined to be 35% based on data from the literature [2, 12]. To reduce the incidence of PONV by 20% in the intervention group at the 5% significance level, the estimated sample size was at least 154 patients, i.e., 77 patients in each group (with a test power of 80% for Pearson’s chi-squared test). Anticipating a 5% dropout rate, we estimated that approximately 162 patients were needed. Shapiro-Wilk test was used to test the normality of distribution of quantitative features. Non-parametric data were analyzed with the Mann-Whitney test. Qualitative characteristics were assessed with the χ^2^ test and Fisher´s exact test, where appropriate. Data are presented as mean (± SD), median [IQR] or counts (percentages). The relative risk of PONV in both groups is presented as risk ratio (RR) and 95% confidence interval (CI). The Stata version 17 program was used for processing the data and *p* < 0.05 was considered statistically significant.

## Results

During the study period, a total of 165 children were enrolled in the clinical trial, of which 2 patients were excluded due to a change in the extent of surgery. 86 children were randomized to the intervention group (BIS-guided anaesthesia), while 77 patients were assigned to the control group (standard anaesthesia practice without BIS monitoring). Baseline patient characteristics, intraoperative vital signs, and duration of anaesthesia and surgery showed no statistically significant differences between the groups. We observed a significant decrease in the consumption of inhalational anesthetic in the intervention group compared to the control group [9.7 (± 1.9) ml vs. 13.2 (± 2.4) ml; *p* = 0.035]. There were no other differences in anaesthesia-related characteristics (Table 1). Throughout the study, there were no cases of unexpected patient movement during surgery requiring deepening of the anaesthesia, and no adverse events were recorded.

**Table 1:** Patient and intraoperative characteristics

	Control (*n* = 77)	BIGA (*N* = 86)	*P* value
Gender (male/female)	49/28	56/30	0.771
ASA status (I/ II)	74/3	81/5	0.629
age (years)	4.8(± 1.3)	4.6(± 1.3)	0.254
weight (kg)	20.3(± 5.5)	18.8(± 5.4)	0.075
height (cm)	111.2(± 10.5)	108.6(± 10.5)	0.064
duration of ga (min)	48.3(± 11.7)	44.8(± 12.3)	0.086
Induction ET _Sev_ (Vol%)	5.8(± 1.3)	5.7(± 1.4)	0.436
ET _Sev_ (Vol%) maintaince of anest.	2.9(± 0.8)	2.2(± 0.7)	0.039^*^
ET _Sev_ (Vol%) end of surgery	2.5(± 0.6)	2.1(± 0.5)	0.047^*^
Consumption of sevoflurane (ml)	13.2(± 2.4)	9.7(± 1.9)	0.035^*^
Sufentanil (µg)	4.1(± 0.2)	4.0(± 0.3)	0.831
Infusion amount (ml)	101.5(± 9.5)	100.8(± 10.1)	0.136
Temperature (°C)	36.3(± 0.1)	36.3(± 0.2)	0.564
Heart rate (beats/min)	115.9(± 14.2)	114.3(± 13.9)	0.621
Respiratory rate (per min)	19.8(± 1.5)	19.7(± 1.4)	0.987
Systolic pressure (MM hg)	95.9(± 11.7)	93.8(± 11.1)	0.812
dIASTOLIC PRESSURE (MM hG)	59.7(± 7.5)	60.1(± 6.9)	0.205
Surgical time (min)	28.1(± 9.1)	30.2(± 11.6)	0.365

The values represent n or mean(± SD). ET_sev_- end tidal concentration of sevoflurane, GA -general anaesthesia. Vital signs were recorded throughout the duration of general anaesthesia.^*^ indicates a statistically significant difference

### Results in the PACU (interval 0–1 h after surgery)

**Fig. 2:**
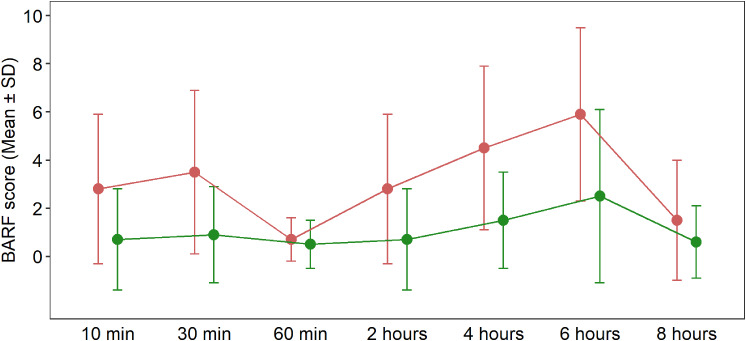
BARF scores in PACU and ward

The incidence of postoperative nausea (PON) was 20% (15/77) in the control group compared to 7% (6/86) in the intervention group (*p* = 0.112). Although the incidence of postoperative vomiting (POV) was not significantly different between groups (*p* = 0.147), patients in the control group required more rescue antiemetic therapy (*p* = 0.043). The rate of postoperative nausea, expressed as BARF score, was significantly improved in the intervention group at 10 and 30 min (*p* = 0.016 and *p* < 0.001) (Table 2; Fig. 2). BIGA led to a significantly less postoperative pain at 10 and 30 min, with lower FLACC score values (*p* = 0.044 and 0.025). FLACC scores at 60 min during the PACU stay were not different between the groups (*p* = 0.076). In the intervention group, rescue analgesic therapy was applied to a significantly smaller number of patients (*p* = 0.003) (Table 2).

**Table 2:** Data recorded in PACU (time interval 0–1 h after surgery)

	Control (*n* = 77)	BIGA (*N* = 86)	*P* value
PACU nausea	15 (20%)	6 (7%)	0.112
PACU vomiting	7 (9%)	3 (4%)	0.147
Number of vomiting episodes	2.1 (± 0.3)	1.3 (± 0.1)	0.042^*^
BARF score 10 min	2 [1-6]	0 [0–2]	0.016^*^
BARF score 30 min	4 [2-8]	0 [0–4]	< 0.001^*^
BARF score 60 min	0 [0–2]	0 [0–2]	0.329
FLACC score 10 min	3 [1-6]	1 [1-3]	0.044^*^
FLACC score 30 min	3 [0–6]	1 [0–3]	0.025^*^
FLACC score 60 min	1 [0–4]	1 [0–3]	0.076
Rescue antiemetic treatment	6 (8%)	1 (1%)	0.043^*^
Rescue analgetic treatment	28 (36%)	14 (16%)	0.003^*^

Data are given as n (%), mean (± SD) or median [IQR]; BARF – Baxter animated retching faces, FLACC - Face, Legs, Activity, Cry, Consolability; ^*^ indicates a statistically significant difference

BARF scores presented as means (SD) during all measured time intervals of intervention group (green color) and control group (red color).

### Results in the ENT department (interval 1–24 h after surgery)

For all children enrolled in this study, the overall incidence of PONV was 34% for nausea (56/163) and 25% (40/163) for postoperative vomiting within the first 24 h after surgery. In the control group a total of 53% (41/77) of patients experienced postoperative nausea with a BARF score above 4 compared to 17% (15/86) in the intervention group (< 0.001). The use of BIS-guided anaesthesia led to a statistically significant decrease in the incidence of PON with risk ratio (RR) = 0.48 (95% CI 0.27–0.86). The incidence of postoperative vomiting was 34% (26/77) in the control group compared with 16% (14/86) in the intervention group (*p* = 0.01). Between the study groups, we found RR = 0.33 (95% CI 0.20–0.54). In cases when the child had already vomited in the ENT department, the number of individual episodes was lower in the intervention group (Table 3). The rate of postoperative nausea, expressed as BARF score, was significantly improved in the intervention group in all measured timepoints (Table 3; Figure 2).

**Table 3:** Data recorded in the ENT ward (time interval 1–24 h after surgery)

	Control (*n* = 77)	BIGA (*N* = 86)	*P* value
Ent ward nausea	41 (53%)	15 (17%)	< 0.001^*^
Ent ward vomiting	26 (34%)	14 (16%)	0.010^*^
Number of vomiting episodes	0.8 (± 1.7)	0.3 (± 0.7)	0.003^*^
BARF score 2 h	2 [2-6]	2 [0–4]	0.022^*^
BARF score 4 h	4 [2-8]	2 [0–6]	< 0.001^*^
BARF score 6 h	6 [2-8]	2 [0–4]	< 0.001^*^
BARF score 8 H	2 [0–4]	0 [0–2]	0.041^*^

Results are represented as n (%);mean (± SD) or median [IQR]; BARF – Baxter animated retching faces; ^*^indicates a statistically significant difference

## Discussion

Despite advances in the prevention and treatment of PONV, it remains one of the most common postoperative adverse events in children [2]. In the present study, we have shown that BIS-guided sevoflurane anaesthesia significantly reduces the incidence of PONV within 1–24 h after the end of surgery compared to standard anaesthesia practice without BIS monitoring. The incidence of early postoperative nausea and vomiting during the PACU stay was not affected, but patients in the non-BIGA group who experienced nausea had higher BARF scores and required more rescue antiemetic treatment. The BIS monitor uses EEG signal processing to measure the depth of the hypnotic component of anaesthesia [13]. BIS values are calculated from 4 EEG sub-parameters: Burst Suppression Ratio (BSR), QUAZI Suppression Index, Relative Beta Ratio (RBR) and SyncFastSlow Ratio (SFS) [14]. Although the device uses an algorithm derived from adult EEG data [15], BIS values also correlate reliably with the depth of sevoflurane anaesthesia in paediatric patients [13, 16].

The administration of inhalational anaesthetics is a strong risk factor for the development of PONV, with the risk increasing with the duration of exposure [4]. Consistent with a previous study [17], we found significantly lower sevoflurane consumption in the BIGA group compared to the non-BIGA group. This resulted in a reduction in the emetogenic effect of sevoflurane with a consequent reduction in the incidence of PONV in the intervention group.

However, we could only show a difference in the incidence of PONV between 1 and 24 h and not in the early postoperative period (0–1 h). These results are in contrast to the Apfel study, which showed a dose-response relationship between volatile anaesthetics and the risk of PONV only in the early postoperative period (0–2 h) [4]. A possible explanation is the use of propofol to treat severe emergence delirium in the PACU, which may have significantly influenced the incidence of early PONV [2], but there was no difference in propofol administration between groups [18]. The lack of effect of BIS-guided anaesthesia in reducing early PONV cannot be explained in our study and warrants further work in this area.

Our results are consistent with a study performed in adult patients in which Nelskylä et al. demonstrated a significant reduction in the incidence of postoperative vomiting by 24% in a group of female patients with BIS-guided depth of general anesthesia. This study also failed to demonstrate a reduction in the incidence of early postoperative vomiting in the PACU [6]. Other works, including two meta-analyses, reported lower but still significant reduction in PONV in BIS-monitored adult patients [8, 19, 20].

In contrast, Liao found no effect of BIS-guided anaesthesia on PONV reduction in children aged 3–12 years undergoing urological surgery [21]. However, this study only included outpatients discharged early from hospital and did not assess the incidence of PONV in the later postoperative period. In addition, the overall incidence of PONV in the PACU was even lower than in our cohort. Another limitation of this study is the unclear methodology used to assess postoperative nausea, which may have led to an underestimation of its prevalence, especially in young children.

Surprisingly, we found greater pain relief in the BIS-guided anaesthesia group. These children had lower levels of postoperative pain, measured by the FLACC score after 10 and 30 min in the PACU, and required less rescue analgesic therapy. The FLACC score at 60 min was similar in both arms of the study, probably because of the higher analgesic consumption in the control group.

Several studies have reported that adequate intraoperative depth of anaesthesia is associated with reduced postoperative pain in adult patients [22–24]. The potential mechanism leading to the reduction of postoperative pain is not entirely clear, but the authors of above-mentioned studies suggested that a higher depth of anaesthesia leads to a partial interruption of nociceptive stimulation with a subsequent lower pain intensity [25]. Our study cannot support this theory, because we did not measure the depth of anaesthesia with the BIS monitor in the control group.

We acknowledge certain limitations in this study. First, this study enrolled patients from a single center. Data from a multicenter study would be more generalisable. The second limitation of this study is the difficulty in assessing postoperative nausea in young children, who in most cases are unable to verbalise this subjective feeling. As a result, the incidence of postoperative nausea may be underestimated. Thirdly, because BIS monitoring was not performed in the control group, we were unable to demonstrate a difference in BIS values between the groups.

## Conclusion

BIS-guided sevoflurane anaesthesia significantly reduces the incidence of nausea and vomiting following surgery in children undergoing adenoidectomy. Future research is needed to futher out study findings in other surgeries and clinical settings.

## Data Availability

The datasets generated and analysed during the current study are available from the corresponding author on reasonable request.

## References

[1] Jin Z, Gan TJ, Bergese SD (2020). Prevention and treatment of postoperative nausea and vomiting (PONV): a review of current recommendations and emerging therapies. Ther Clin Risk Manag.

[2] Kovac AL (2020). Postoperative nausea and vomiting in pediatric patients. Pediatr Drugs.

[3] Gan TJ, Belani KG, Bergese S, Chung F, Diemunsch P, Habib AS (2020). Fourth Consensus guidelines for the management of postoperative nausea and vomiting. Anesth &Analgesia.

[4] Apfel CC, Stoecklein K, Lipfert P, Ponv (2005). A problem of inhalational anaesthesia? Best practice. Res Clin Anaesthesiol.

[5] Apfel CC, Kranke P, Katz MH, Goepfert C, Papenfuss T, Rauch S (2002). Volatile anaesthetics may be the main cause of early but not delayed postoperative vomiting: a randomized controlled trial of factorial design †. Br J Anaesth.

[6] Nelskylä KA, Yli-Hankala AM, Puro PH, Korttila KT (2001). Sevoflurane titration using Bispectral index decreases postoperative vomiting in phase II recovery after ambulatory surgery. Anesth &Analgesia.

[7] Ferreira AL, Mendes JG, Nunes CS, Amorim P (2019). Evaluation of Bispectral Index time delay in response to anesthesia induction: an observational study. Brazilian J Anesthesiology.

[8] Luginbühl M, Wüthrich S, Petersen-Felix S, Zbinden AM, Schnider TW (2003). Different benefit of Bispectal Index (BISTM) in Desflurane and propofol anesthesia. Acta Anaesthesiol Scand.

[9] Eberhart LH, Geldner G, Kranke P, Morin AM, Sch??uffelen A, Treiber H, et al. The development and validation of a risk score to predict the probability of postoperative vomiting in pediatric patients. Anesth Analgesia. 2004;1630–7. 10.1213/01.ane.0000135639.57715.6c. 10.1213/01.ANE.0000135639.57715.6C 15562045

[10] Watcha MF, Lee AD, Medellin E, Felberg MT, Bidani SA (2019). Clinical use of the Pictorial Baxter Retching faces scale for the measurement of postoperative nausea in children. Anesthesia&Analgesia.

[11] Merkel S, Voepel-Lewis T, Malviya S (2002). Pain assessment in infants and young children: the FLACC scale. AJN. Am J Nurs.

[12] Frelich M, Divák J, Vodička V, Masárová M, Jor O, Gál R (2018). Dexamethasone reduces the incidence of postoperative nausea and vomiting in children undergoing endoscopic adenoidectomy under general anesthesia without increasing the risk of postoperative hemorrhage. Med Sci Monit.

[13] Denman WT, Swanson EL, Rosow D, Ezbicki K, Connors PD, Rosow CE (2000). Pediatric evaluation of the Bispectral Index (BIS) monitor and correlation of BIS with End-tidal sevoflurane concentration in infants and children. Anesth Analgesia.

[14] Lee HC, Ryu HG, Park Y, et al. Data Driven Investigation of Bispectral Index Algorithm. Sci Rep. 2019;9(1). 10.1038/s41598-019-50391-x. 10.1038/s41598-019-50391-x PMC6760206 31551487

[15] Rampil IJ (1998). A primer for EEG Signal Processing in Anesthesia. Anesthesiology.

[16] Degoute C-S, Macabeo C, Dubreuil C, Duclaux R, Banssillon V (2001). EEG bispectral index and hypnotic component of anaesthesia induced by sevoflurane: comparison between children and adults. Br J Anaesth.

[17] Bannister CF, Brosius KK, Sigl JC, Meyer BJ, Sebel PS (2001). The Effect of Bispectral Index Monitoring on Anesthetic Use and Recovery in Children anesthetized with sevoflurane in Nitrous Oxide. Anesth Analgesia.

[18] Frelich M, Lečbychová K, Vodička V, Ekrtová T, Sklienka P, Jor O (2024). Effect of bis-guided anesthesia on emergence delirium following general anesthesia in children: a prospective randomized controlled trial. Anaesth Crit Care Pain Med.

[19] Oliveira CR, Bernardo WM, Nunes VM (2017). Benefit of general anesthesia monitored by BISPECTRAL index compared with monitoring guided only by clinical parameters. Systematic review and meta-analysis. Brazilian J Anesthesiology (English Edition).

[20] Liu SS (2004). Effects of BISPECTRAL index monitoring on ambulatory anesthesia. Anesthesiology.

[21] Wen W, Liao JJ, Wang, Wu GJ, Cheng Deng, Kuo (2011). The effect of cerebral monitoring on recovery after sevoflurane anesthesia in ambulatory setting in children: a comparison among bispectral index, A-line autoregressive index, and standard practice. J Chin Med Association.

[22] Henneberg SW, Rosenborg D, Weber Jensen E, Ahn P, Burgdorff B, Thomsen LL (2005). Peroperative depth of anaesthesia may influence postoperative opioid requirements. Acta Anaesthesiol Scand.

[23] Soumpasis I, Kanakoudis F, Vretzakis G, Arnaoutoglou E, Stamatiou G, Iatrou C (2010). Deep anaesthesia reduces postoperative analgesic requirements after major urological procedures. Eur J Anaesthesiol.

[24] Gurman GM, Popescu M, Weksler N, Steiner O, Avinoah E, Porath A (2003). Influence of the cortical electrical activity level during general anaesthesia on the severity of immediate postoperative pain in the morbidly obese. Acta Anaesthesiol Scand.

[25] Anand L, Sahni N, Gombar K, Gombar S (2011). Effect of intraoperative depth of anesthesia on postoperative pain and analgesic requirement: a randomized prospective Observer Blinded Study. J Anaesthesiol Clin Pharmacol.

